# Supplementary material for: miR-21-3p/IL-22 Axes Are Major Drivers of Psoriasis Pathogenesis by Modulating Keratinocytes Proliferation-Survival Balance and Inflammatory Response
Source: Cells. 2021 Sep 26;10(10):2547. doi: 10.3390/cells10102547 (PMC8534095; doi:10.3390/cells10102547)
Supplement: Supplementary file 1 [file cells-10-02547-s001.zip › Supplemental data.pdf]

Supplemental Methods

Western Blot

Cells were lysed with RIPA buffer (Thermo Scientific #89901) supplemented with 1% Protease inhibitor cocktail (Sigma, P8340), 1%PMSF (Sigma P-7626), 50mM NaF (Sigma S1504). Total protein amount quantification was achieved by BCA protein assay kit (Interchim, protein quantitation kit, BCAssays, UP40840A). 10µg of protein lysates from HaCaT cells were separated by SDS-PAGE 10% gel, blotted onto PVDF membranes, and probed with rabbit anti-human Cyclin-D1 antibody (1/200, sc-753). As loading control, the blots were probed using human β-actin (1/20000, Sigma-Aldrich, A2066). Bound antibodies were revealed using horseradish peroxidase (HRP)-conjugated goat anti-rabbit antibody (1/1000; ab97051) and detected using enhanced chemiluminescence (ECL) reagents. The Trans-Blot Turbo RTA transfer Kit, LF PVDF was used (BioRad, #170-4275).

Histopathological analysis

After treatment of mice with IMQ, the mouse back skin was fixed in formalin and embedded in paraffin. Sections of 6 µm were stained with Hematoxylin (Sigma #MHS80) and Eosin (Sigma #HT110232).

Supplementary figures

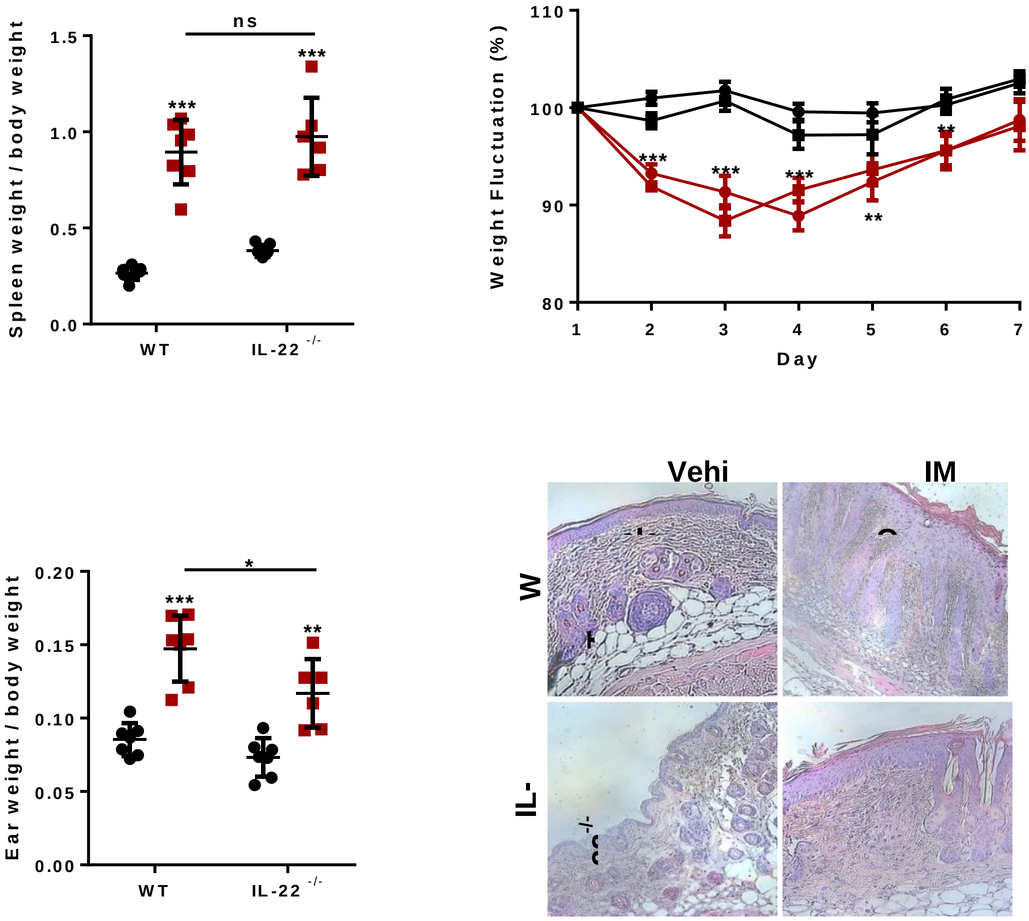

Figure S1. Imiquimod mice model Wild-Type versus IL-22 deficient mice. Follow up of the pathogenesis development throughout the 7 days model by visual inspection represented by mice photography (A), a score scale evaluating the redness and scaling of the skin (B), the spleen over body weight ratio at day 7 (C), the histological features of the skin by H&E staining of skin biopsies (D), evolution of the body weight (E) and ear over body weight evaluation at day 7 (F). Two-way ANOVA test: *P<0.05, **P<0.01, ***P<0.001. Results are presented, as mean values ±SEM.

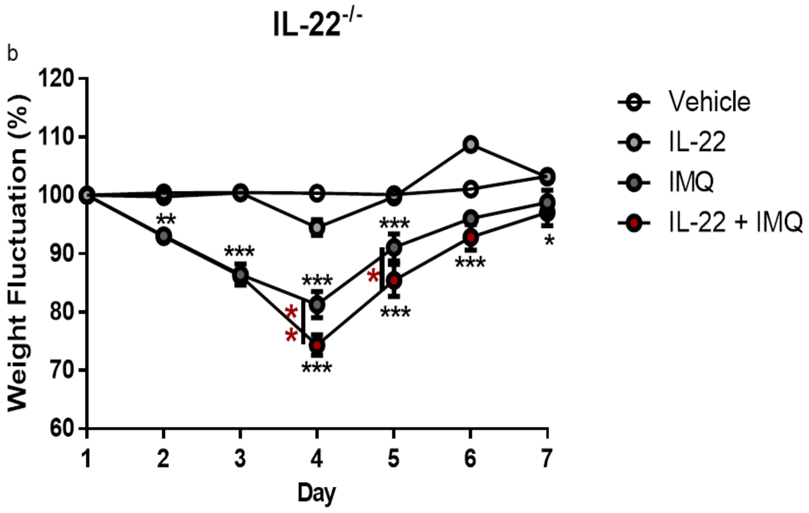

Figure S2. Body weight fluctuation in IL-22 treated IL-22 deficient mice. IMQ induced psoriasiform in IL-22-/- mice that received subcutaneously 100ng of IL-22 or equivalent of physiological serum on the shaved, one day before pathogenesis induction and up to 7 days. Data presented from one experiment with n=5. Two-way ANOVA test (c): *P<0.05, **P<0.01, ***P<0.001. Data are from 6 samples (mean and SEM).

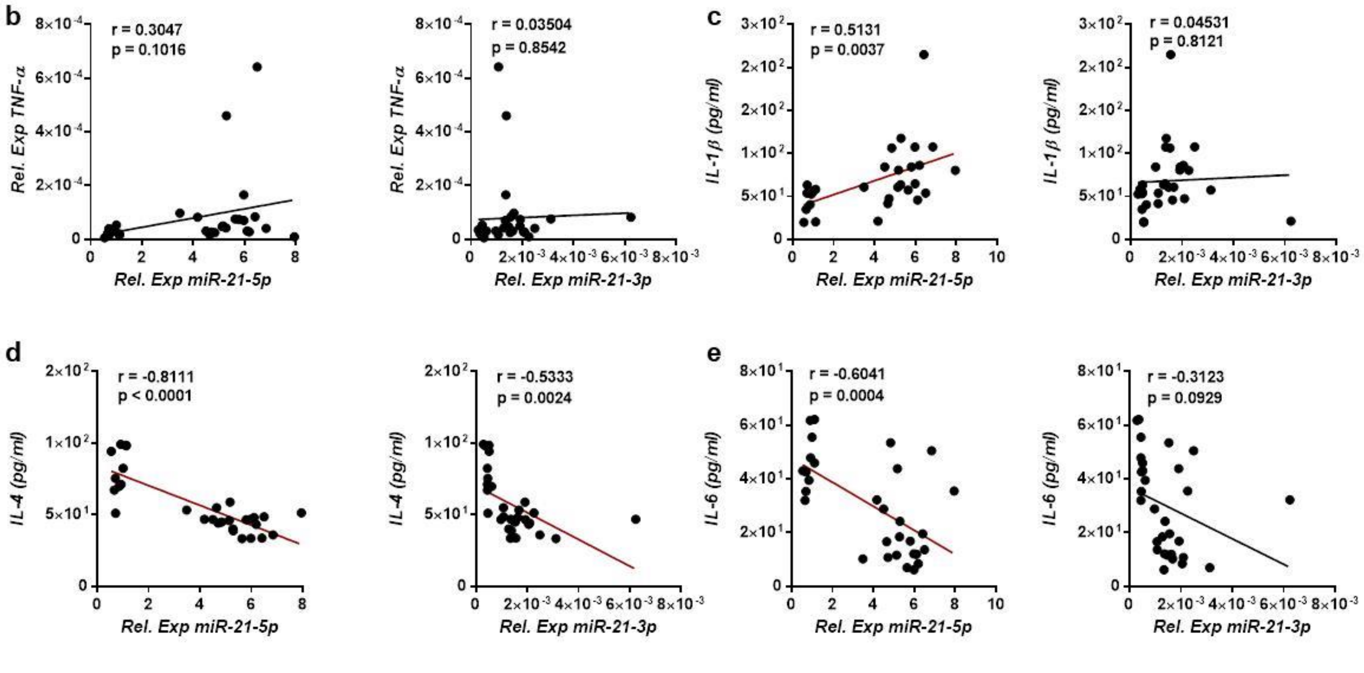

Figure S3. miR-21-5p and 3p expression correlation with inflammatory mediators. Pearson correlations between miR-21-5p or miR-21-3p and mRNA IL-22 or protein IL-22 (A), mRNA TNF-α (B), protein IL-1β (C), protein IL-4 (D), protein IL-6 (E) were established in 30 mice psoriatic skin (E). mRNA expression was evaluated by RT. qPCR whereas protein expression was assessed by ELISA. Data are representative of two independent experiments with five mice per group. *P<0.05, **P<0.01, ***P<0.001. Results are presented, as mean values ±SEM.

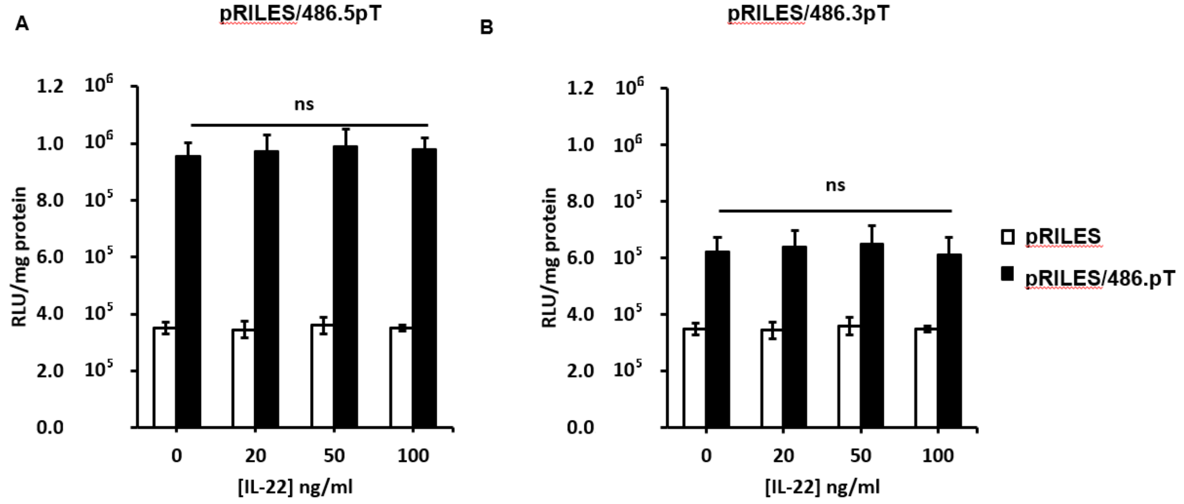

Figure S4. Functional assay using RILES (RNAi - Inducible Luciferase Expression System) experimental control in HaCaT cells. miR-486-5p (A) and miR-486-3p (B) expression induction assessment using RILES technology in HaCaT cells stimulated with 0, 20, 50 and 100ng/ml of IL-22 for 24 hours. Data are presented as a Relative luciferase unit (RLU) normalized to the total amount of protein (mg).

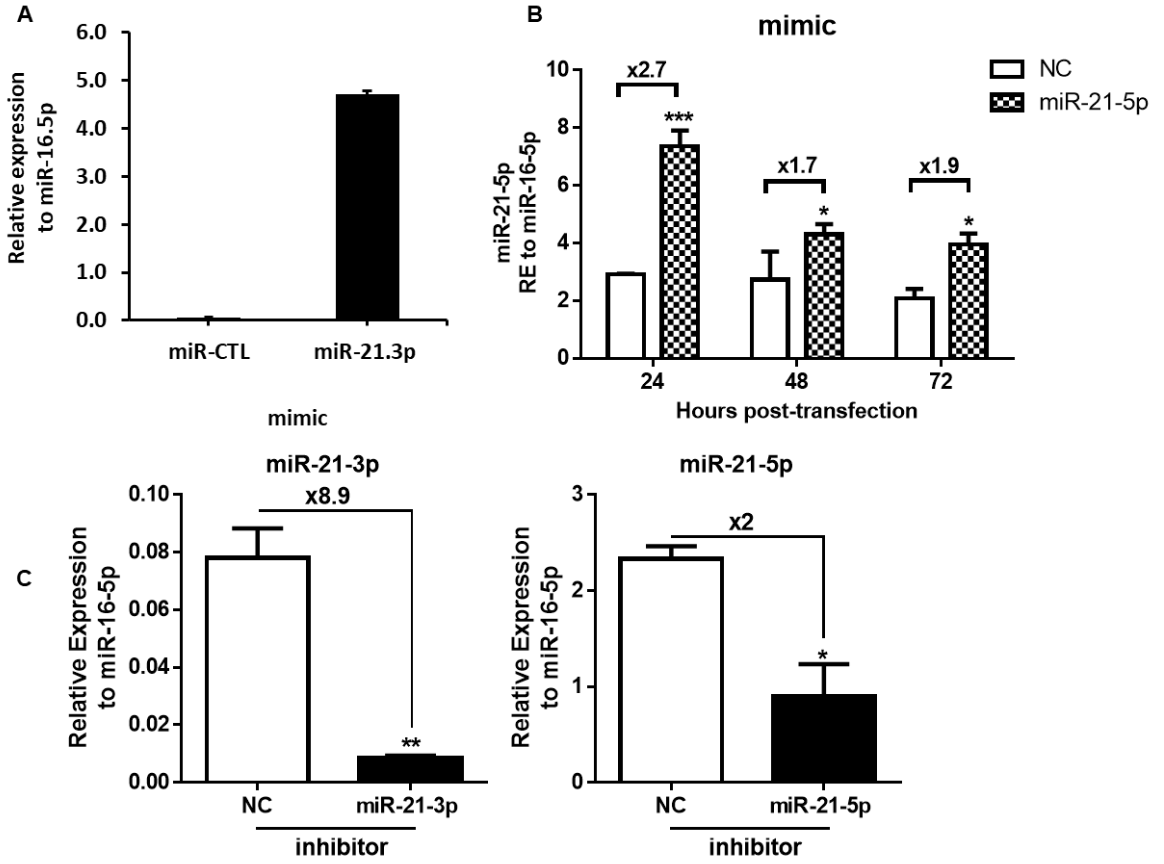

Figure S5. Transfection efficiency. The transfection efficiency was evaluated by RTqPCR for 10nM transfected HaCaT cells with Negative Control mimic (miR-Ctrl)/miR-21-3p mimic 24h post-transfection (A) or miR-21-5p mimic at 24h/48h/72h post-transfection (B), with Negative Control inhibitor (NC) / miR-21-3p inhibitor (C) or miR-21-5p inhibitor

(D) 24h post-transfection. Two way ANOVA test or t-test: *P<0.05, **P<0.01, ***P<0.001. Data are from two independent experiments done in triplicate (mean and SEM).

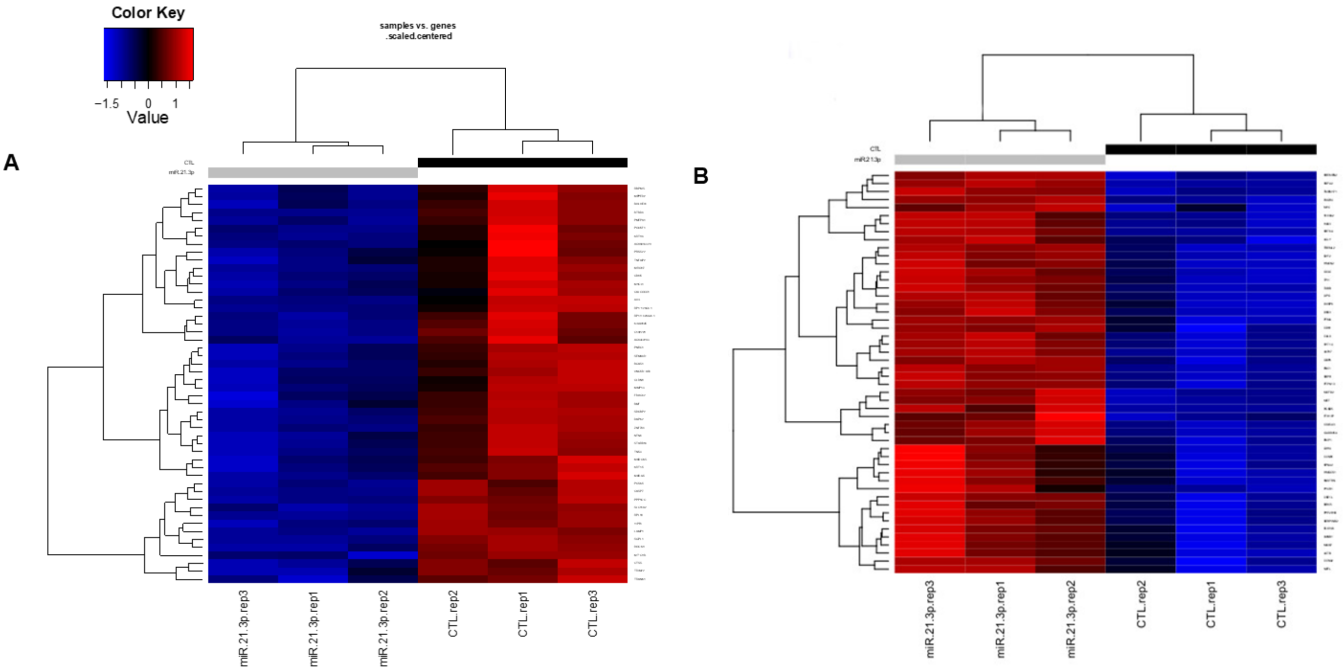

Figure S6. Heat Map of DEGs in miR-21-3p overexpressing KCs versus NC. Heatmaps showingthe top 50 up-regulated (A) and down-regulated genes (B) in miR-21-3p versus. NC with a p.adjusted <0.05 and the Pearson correlation between samples respectively.

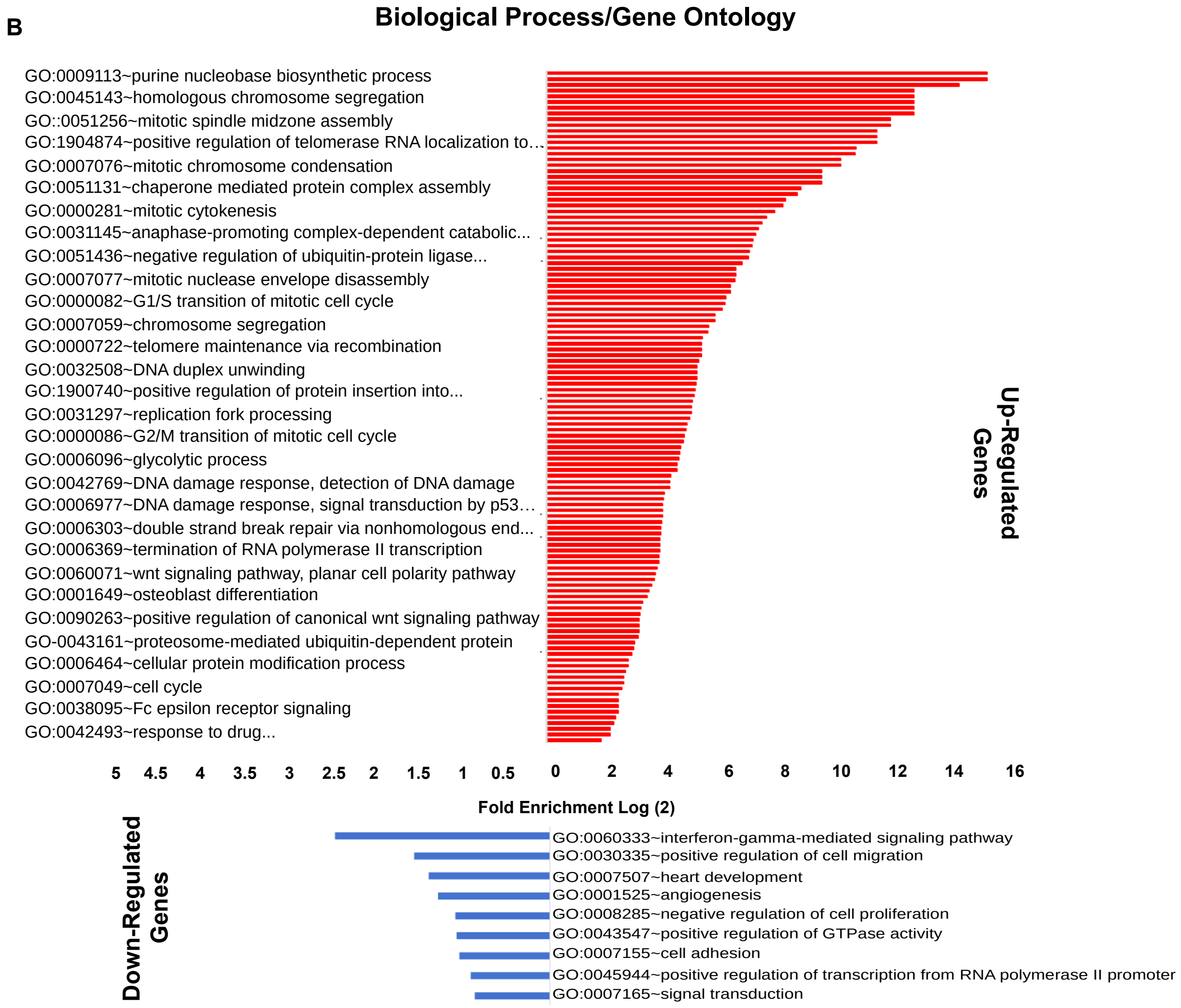

Figure S7. Relevant biological processes (gene ontology) from Gene Set Enrichment Analysis (David) of genes induced by DEGs in miR-21-3p overexpressing KCs versus NC. Red bars denote positive enrichment (i.e. genes disproportionally induced), while blue bars denote negative enrichment (i.e. genes disproportionally repressed).

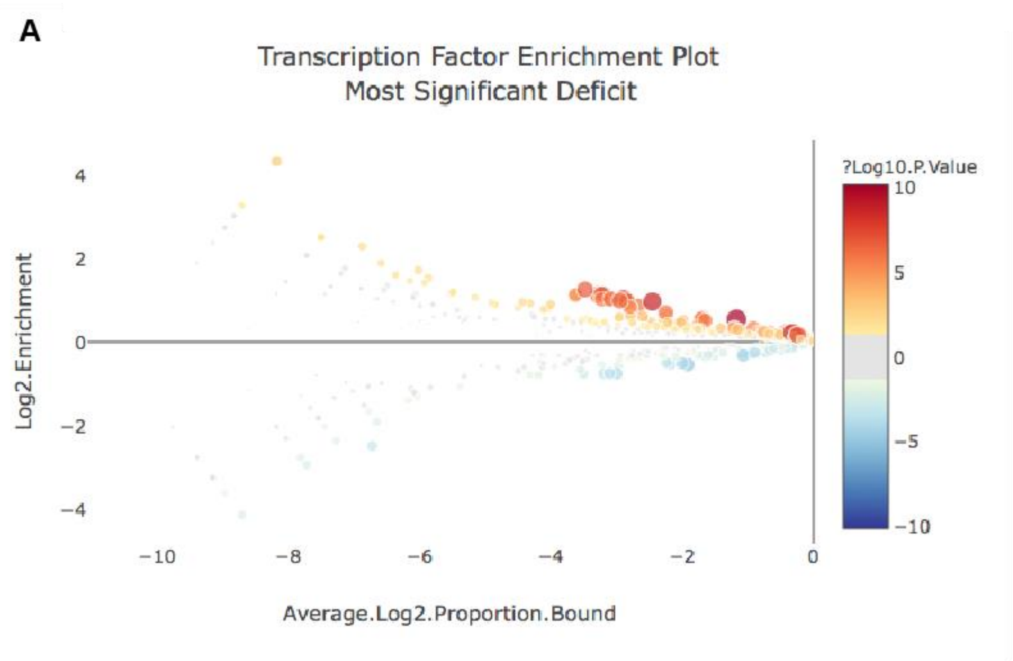

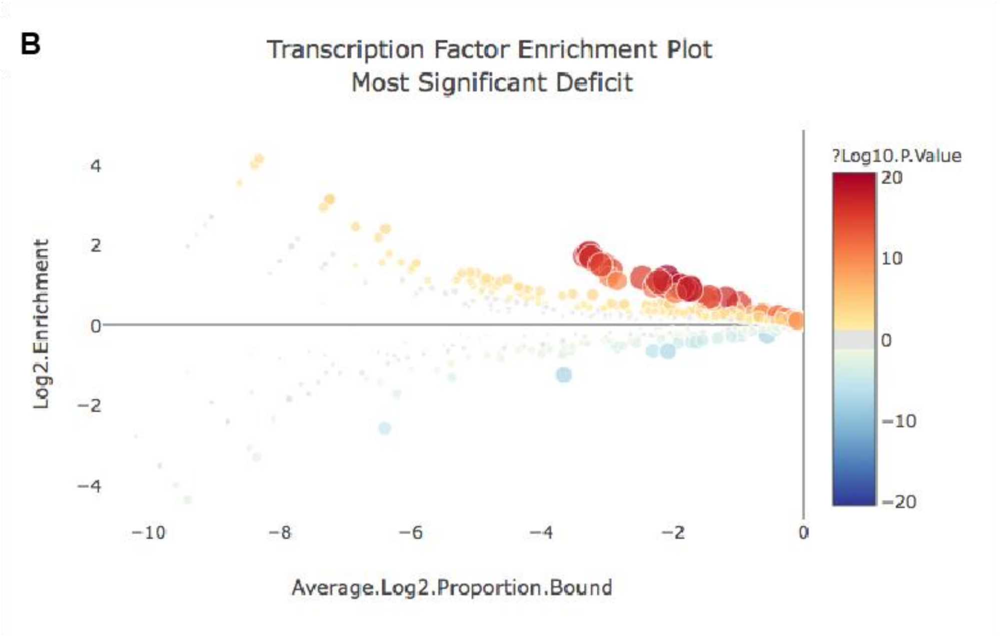

Figure S8. Transcription factor enrichment plot. Proximal transcription factor enrichments (<=600bp), performed by Ci-ider 0.9 with the Jaspar matrix, of genes upregulated (A) and downregulated (B) in miR-21-3p overexpressing KCs versus NC. Background genes were human genes don't include in the respective DEGs set.

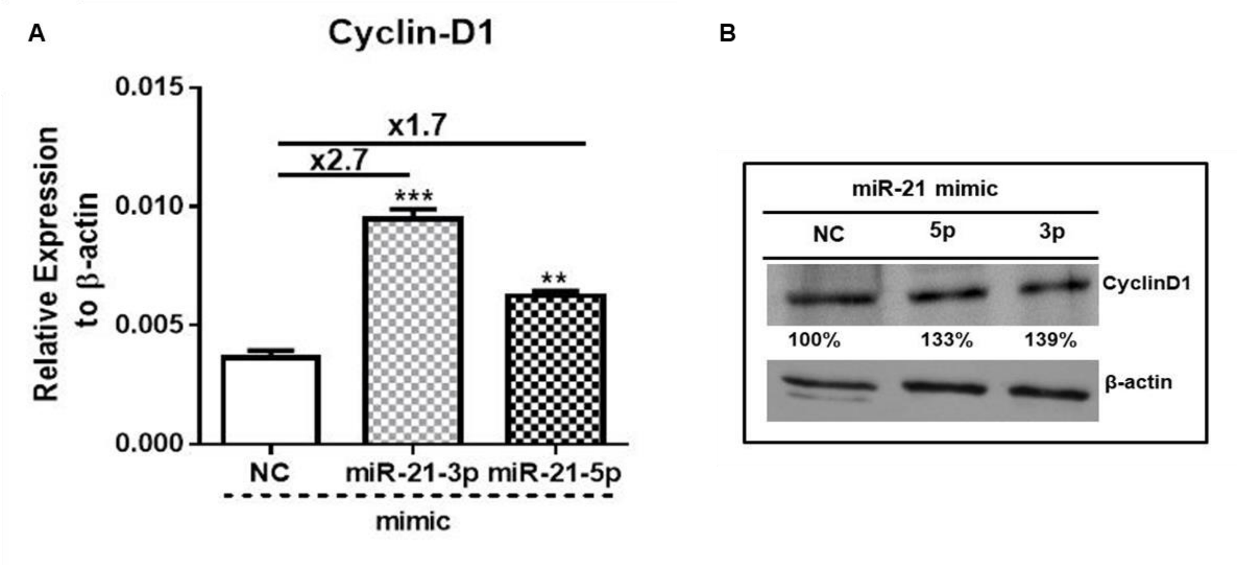

Figure S9. RT qPCR analysis for Cyclin D1 relative expression (A) WB of CyclinD1 normalized on β-actin (B) from HaCaT cells transfected with 10nM Negative control (NC), miR-21-5p and miR-21-3p mimics in steady state. Results of WB are representative of 1 over 2 independent experiments. Two-way ANOVA test: *P<0.05, **P<0.01, ***P<0.001. Data are from three independent experiments done in triplicate (mean and SEM).

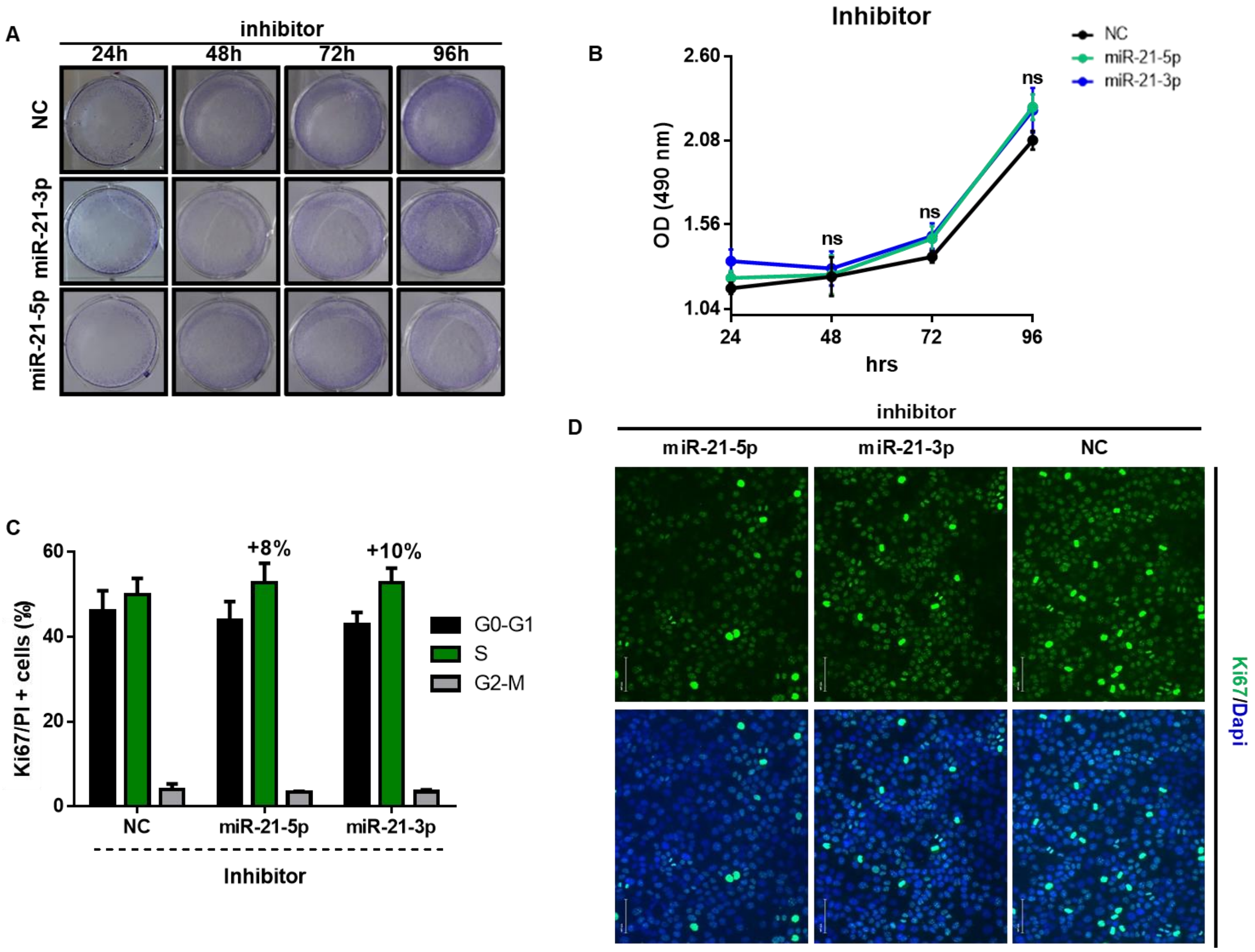

Figure S10. miR-21-3p loss of function impact on KCs proliferation. (A) Crystal violet staining at different time point (24h, 48, 72h and 96h) of HaCaT cells transfected with negative control (NC), miR-21-5p or miR-21-3p inhibitor. (B) XTT assay at different time point (24h, 48, 72h and 96h) of HaCaT cells transfected with negative control (NC), miR-21-5p or

miR-21-3p inhibitors (C) Cell cycle analysis with double Ki-67/PI staining 24h post-transfection of HaCaT cells with negative control (NC), miR-21-5p or miR-21-3p inhibitors. (D) Ki67 immunostaining 24h post transfection of HaCaT cells with negative control (NC), miR-21-5p or miR-21-3p inhibitors. Data information: Results are presented, as mean values ±SEM. Data are representative of three independent experiments performed in triplicates. The statistical comparison between groups was performed by using Two-way ANOVA test: $*P<0.05$, $**P<0.01$, $***P<0.001$.
